# View from the Top: Insights into the Diversity and Community Assembly of Ectomycorrhizal and Saprotrophic Fungi along an Altitudinal Gradient in Chinese Boreal *Larix gmelinii*-Dominated Forests

**DOI:** 10.3390/microorganisms10101997

**Published:** 2022-10-10

**Authors:** Yi Guo, Li Ji, Mingwei Wang, Chengfeng Shan, Fangyuan Shen, Yuchun Yang, Gongxiu He, Witoon Purahong, Lixue Yang

**Affiliations:** 1 School of Forestry, Central South University of Forestry and Technology, Changsha 410004, China; 2 Key Laboratory of Sustainable Forest Ecosystem Management-Ministry of Education, School of Forestry, Northeast Forestry University, Harbin 150040, China; 3 Department of Soil Ecology, UFZ-Helmholtz Centre for Environmental Research, 06120 Halle (Saale), Germany; 4 Jilin Academy of Forestry, Changchun 130033, China

**Keywords:** ectomycorrhizal fungi, saprotroph, altitudinal gradient, community assembly, *Larix gmelinii*

## Abstract

The altitudinal patterns of soil fungi have attracted considerable attention; however, few studies have investigated the diversity and community assembly of fungal functional guilds along an altitudinal gradient. Here, we explored ectomycorrhizal (EcM) and saprotrophic (SAP) fungal diversity and community assembly along a 470 m vertical gradient (ranging from 830 to 1300 m) on Oakley Mountain, sampling bulk soils in the 0–10 cm and 10–20 cm soil layers of *Larix gmelinii*-dominated forests. Illumina MiSeq sequencing of the ITS genes was employed to explore the fungal community composition and diversity. The relative abundance of EcM and SAP fungi showed a divergent pattern along an altitudinal gradient, while we observed a consistent altitudinal tendency for EcM and SAP fungal diversity and community assembly. The diversity of both fungal guilds increased with increasing altitude. Altitude and soil moisture were the key factors affecting the community composition of both fungal guilds. In addition, the plant community composition significantly affected the EcM fungal community composition, whereas the dissolved organic nitrogen and ammonium nitrogen contents were the driving factors of SAP fungal community. Despite the effects of vegetation and soil factors, EcM and SAP fungal communities were mainly governed by stochastic processes (especially drift) at different altitudes and soil depths. These results shed new light on the ecology of different fungal functional guilds along an altitudinal gradient, which will provide a deeper understanding of the biogeography of soil fungi.

## 1. Introduction

Fungi play a critical role in forest soils; they are responsible for carbon reservoir and nutrient cycling, and thus interact with host plants in ways based on the different lifestyle ranges [1]. The development of high-throughput sequencing techniques provides future avenues for research on fungal biogeography, allowing the high-efficiency analysis of the diversity patterns of fungi across varied habitats and scales [2,3]. Altitude is a comprehensive environmental gradient, and exhibits a heterogeneity of abiotic and biotic conditions that remarkably affect the spatial variation of soil microbiotas [4]. Although previous studies have primarily focused on fungal diversity and community composition as a whole along an altitudinal gradient, fungal functional guilds have been less studied, despite new molecular approaches progressing the topic [5,6].

Ectomycorrhizal (EcM) and saprotrophic (SAP) fungi are the two main fungal functional groups and are associated with functionally different lifestyles associated with litter decomposition and nutrient cycles [7,8]. As a prevailing guild of fungi in boreal and temperate forests, EcM fungi acquire resources by symbiotically interacting with both the host and the surrounding environment (abiotic conditions) [9,10]. Additionally, saprobes are free-living fungi capable of degrading complex organic molecules (e.g., lignin, cellulose and hemi-cellulose) by producing powerful enzymes, acting as major decomposers in forest ecosystems [11]. By releasing nutrients from dead organisms, these saprotrophic fungi provide support for the vital process of nutrient cycling, as well as participating in the global carbon cycle [7]. Recently, the characteristics of EcM and SAP fungal communities along altitudinal gradients have received more attention, and been the subject of inconsistent observations [12,13]. Gong et al. [14] suggested that the EcM fungal richness in soil and roots declined in the forest zone but increased in the alpine meadow zone. In contrast, Yang et al. [12] found that the EcM fungal diversity of *Betula ermanii* was the lowest at high-elevation sites in Mt. Changbai, and the diversity of SAP fungi was the best predictor of this. Miyamoto et al. [15] found an obvious mid-domain effect of EcM fungi at Mount Fuji, and elevation and host plants account for more of the variation in EcM species. As EcM and SAP fungi produce extracellular enzymes to degrade complex polymers and release available nutrients to maintain growth and metabolic functions, they both rely on the litter for survival [16,17]. Moreover, SAP fungi can be inhibited by EcM fungi due to their competition for nitrogen, water or space [18,19], and earlier hypotheses suggested that this competition could slow down decomposition rates (i.e., the “Gadgil effect”) [20,21]. Thus, more competition between EcM and SAP fungi may be observed in harsh and adverse areas [22], where a low decay rate is caused by active extracellular enzymes and nitrogen limitation in the case of cold-adapted decomposers [23]. Shigyo and Hirao [13] reported that SAP and EcM fungal richness are affected by soil properties and climate factors in cool-temperate montane forests in Japan. However, much less is known about the difference in distribution patterns of EcM and SAP fungi along an altitudinal gradient in boreal forests.

As hosts and symbionts migrate, altered interactions between plants and fungal species, and changes in the niche differentiation related to the carbon cycle, can induce distinct shifts in EcM and SAP fungal communities [24,25]. Unraveling the mechanism of the community assembly of soil microbiota has become a central issue related to distribution patterns along environmental gradients [26,27,28]. While some researchers have reported that determinism and stochasticity govern fungal community assemblages in a variety of habitats [29,30,31], to date, there is still very little scientific evidence for either of the two key fungal guilds (EcM and SAP fungi). Gao et al. [32] demonstrated that the EcM fungal community was mainly controlled by environmental selection and dispersal limitation at different stages of secondary forest succession. Matsuoka et al. [33] found that in addition to the driving effect of host trees and environmental variables along an altitudinal gradient, the EcM fungal community was mediated by the stochastic dispersal processes. Therefore, deciphering the relative contribution of deterministic and stochastic processes in EcM and SAP fungal community will shed light on the potential mechanism of the niche differentiation of fungal guilds.

Oakley Mountain is the highest peak in the northern Greater Khingan Mountains, with the characteristics of low average annual temperature and a short freezing period. The coniferous forests dominated by *Larix gmelinii* are an important component of the boreal forest ecosystem in this region [34]. Mt. Oakley provides a natural and ideal platform for exploring the altitudinal patterns of the plant community, edaphic conditions, and microbial community structures [34]. Our recent studies on Mt. Oakley revealed that altitude has a stronger effect on fungal community composition than season and soil depth, and fungal interactions and keystones exhibit obvious seasonal succession [34,35]. However, far too little attention has been paid in previously published studies to the diversity and community composition of fungal functional guilds, especially community assembly processes. In this study, we examined EcM and SAP fungal community structures in bulk soils of the *Larix gmelinii-*dominated natural forests along a 470 m altitudinal gradient of Mt. Oakley, China. The aim is to describe the diversity, community composition and assembly of EcM and SAP fungi along an altitudinal gradient and reveal the causal mechanisms of both key fungal guilds in boreal forests ecosystems. Because of the limiting nutrients, the high proportion of coniferous trees and the harsh environment at high altitude, we hypothesized that: (1) The relative abundance and diversity of EcM fungi will increase with increasing altitude, whereas we will observe a contrary tendency in SAP fungi. In addition, as we found an altitudinal pattern in a fungal community in a previous study [35], altitude will have a stronger effect on EcM and SAP community composition than soil depth. (2) Given the well-differentiated resource acquisition strategies, as well as the sharing of common ancestors [21], the EcM fungal community composition will be affected by the plant community (host plant), while we predicted that SAP fungi will be more driven by the soil nutrient contents. (3) Additionally, the EcM fungal community will be dominated by deterministic processes, whereas SAP fungi will be mediated by stochasticity.

## 2. Materials and Methods

### 2.1. Site Description and Soil Sampling

The study area is located at the A’longshan Forestry Bureau in Inner Mongolia, China (Figure S1). Oakley Mountain is the highest peak of the northern area of the Greater Khingan Mountain, which is characterized by a cold-temperate climate with short summers and long winters. The average annual temperature and average annual precipitation are −5.1 °C and 437.4 mm, respectively. The elevation ranges from 600 m to 1520 m a.s.l. The vegetation type is a boreal coniferous forest that consists primarily of *Larix gmelinii*, *Betula platyphylla*, and *Pinus sylvestris* arbor trees, and *Rhododendron dauricum* and *Spiraea dahurica* shrubs. As one of the dominant forest species on Mt. Greater Khingan, *Larix gmelinii* is naturally distributed across all altitudes in this region. The soils of the larch forests are Umbric Cryosols and Gelic Podzols, with a depth of 20–25 cm on average.

In September 2018, based on the criterion of different vegetation compositions or tree lines, four altitude sites of 830 m, 950 m, 1100 m and 1300 m were selected, and three independent replications (30 × 30 m) were conducted at each altitude. Nine soil cores from each experimental plot were randomly collected from the 0–10 cm and 10–20 cm soil layers after removing the litter. One composite sample was created by pooling and homogenizing all soil samples from the same plot and immediately transported in an icebox to the laboratory. A 2 mm mesh was used to sieve all of the fresh soil samples, and visible roots and gravel were discarded. All samples were subdivided into two subsamples. For edaphic parameters, subsamples were stored at 4 °C, and subsamples for molecular analysis were kept at −80 °C. The detailed information is derived from the descriptions of Ji et al. [35].

### 2.2. Soil Property Analysis

The soil pH and bulk density (BD) were determined with a digital pH meter (MT-5000, Sanbon, Shanghai, China) and the cutting ring method, respectively. Using a J200 Tandem laser spectroscopic Element Analyzer (Applied spectrum, Inc., Fremont, CA, USA), the soil organic carbon (SOC) and total nitrogen (TN) contents were measured. The total phosphorus (TP) content was digested using hydrofluoric acid and perchloric acid and determined by molybdenum blue colorimetry (TU-1901, Puxi Ltd., Beijing, China). The soil dissolved organic carbon (DOC) and dissolved total nitrogen (DTN) were determined with a total organic carbon analyzer (Analytik Jena, multi N/C 3000, Jena, Germany). The soil nitrate nitrogen (NO_3_^−^-N) and ammonium nitrogen (NH_4_^+^-N) were determined by the continuous flow analytical system (AA3, Seal Co., Norderstedt, Germany). The soil dissolved organic nitrogen (DON) was calculated from The NO_3_^−^-N, NH_4_^+^-N, and DTN contents of the soil were used to calculate the dissolved organic nitrogen (DON) content. The chloroform fumigation method was used to determine the carbon, nitrogen, and phosphorus (MBC, MBN, and MBP) in the soil microbial biomass [36].

### 2.3. Soil Fungal DNA Extraction and Polymerase Chain Reaction Amplification

Using an E.Z.N.A.^®^ Soil DNA Kit (Omega Biotek, Norcross, GA, USA), fungal DNA was extracted from the soil samples in accordance with the instructions provided by the manufacturer. Using a NanoDrop 2000 spectrophotometer (Thermo Scientific, Wilmington, NC, USA), the DNA extracts were then mixed together and quantified on a 1.0% (*w/v*) agarose gel. The fungal ITS genes were amplified. The primers ITS3F (5′-GCATCGATGAAGAACGCAGC-3′) and ITS4R (5′-TCCTCCGCTTATTGATATGC-3′) were employed to amplify the ITS2 region. The fungal primers were derived with a thermocycler PCR system (GeneAmp 9700, ABI, USA). A detailed description of the PCR amplification was reported in our previous study [35].

### 2.4. Illumina MiSeq Sequencing and Bioinformatic Analyses

The PCR products were extracted with a 2% agarose gel, pooled with equimolar amounts and then purified using the AxyPrep DNA Gel Extraction Kit (Axygen Biosciences, Union City, CA, USA). Using a QuantiFluorTM-ST fluorometer (Promega, Madison, WI, USA), the products were quantified in accordance with the manufacturer’s protocols. According to Majorbio Bio-Pharm Technology Co., Ltd.’s standard protocols, the amplified fragments were then paired-end sequenced (2 × 300 bp) and merged on the Illumina MiSeq platform (Illumina, San Diego, CA, USA). The original sequencing data have been deposited in the National Center for Biotechnology Information Sequence Read Archive (SRA) database under BioProject ID: PRJNA721105.

Trimmomatic was conducted to demultiplex the raw fastq files, and quality filtering was carried out, and FLASH was used to merge the reads [37]. Based on their 97% similarity, the sequences were grouped into operational taxonomic units (OTUs) using UPARSE (version 7.1, http://drive5.com/uparse/, accessed on 10 April 2022) [38]. UCHIME was used to identify chimeric sequences. Singletons and chimeras were eliminated. Using the UNITE fungal ITS databases, the ITS gene sequences’ taxonomic identities were determined by BLAST. The FUNGuilds database was used to assign fungal functional guilds based on the OTU table and fungal Genus-level taxonomy annotations [5,39]. Due to the annotation depending on life stage and inhabiting conditions, some fungi did not fall exclusively into a single guild; thus, EcM and SAP fungi with a confidence ranking “highly probable” were assigned in the current analysis as recommended by the authors (http://www.stbates.org/guilds/app.php, accessed on 10 April 2022) [5]. A total of 2739 fungal OTUs were found across all the soil samples, but only half of them (1457 OTUs) were assigned by FUNGuild.

### 2.5. Statistical Analyses

To estimate the alpha diversity of fungal communities, the observed number of OTUs, Shannon and Chao 1 indices were calculated based on the OTU tables in QIIME. Two-way ANOVA was conducted to test the effect of altitude and soil depth on EcM and SAP fungal community characteristics. Tukey’s honestly significance test was used to test significant differences among different altitudes at *p* < 0.05, while the *T*-test was performed to test the significant differences between contrasting soil depths. All data were checked for normality and the homogeneity of variance.

Using the “pheatmap” package in R, the relative abundances of the top 50 most abundant classified EcM and SAP fungal OTUs were compared between treatments using a heat map analysis (Version 4.0.5, The University of Auckland, Auckland, New Zealand). The shared and unique OTUs among treatments were counted, and their distributions are shown in a Venn diagram created with the “VennDiagram” package in “R”. Using the “vegan” package in the R software (v4.0.5, The University of Auckland, Auckland, New Zealand), the nonmetric multidimensional scaling (NMDS) ordinations based on Bray–Curtis dissimilarity matrices were used to analyze the β-diversity of the EcM and SAP fungal communities. Permutation multivariate analysis of variance (PERMANOVA) was employed to test for differences in community similarity between altitude and soil depth using the “adonis” function of the “vegan” package in R software. The value of *R*^2^ denotes the percentage of the variance explained by the groups. The main drivers that were significantly correlated with the soil bacterial and fungal communities based on Spearman’s correlation were evaluated using a partial Mantel test with 999 permutations (*p* < 0.05) using the vegan package in R.

The phylogenetic normalized stochasticity ratio (pNST) was performed to quantify the relative contribution of deterministic and stochastic processes in community assembly. The beta nearest taxon indices (βNTI) and Raup–Crick (RC_bray_) null model based on Bray–Curtis dissimilarity were further conducted to quantify dispersal-based stochastic ecological processes. For pNST index, 50% was set as the boundary point between more deterministic (pNST < 50%) and more stochastic (pNST > 50%) assembly. The relative proportion of dispersal limitation and homogenizing dispersal processes was assessed by |βNTI| < 2, RCbray > +0.95 and RCbray < −0.95, respectively, and the undominated process was estimated by |βNTI| < 2 and |RCbray| < 0.95. The detailed descriptions refer to the study of Ji et al. [40]. All the parameters above mentioned were calculated by using the “iCAMP” package in R with the code provided by Ning et al. [41] (https://github.com/DaliangNing/iCAMP1; accessed on 10 April 2022).

## 3. Results

### 3.1. General Descriptions of EcM and SAP Fungi

After bioinformatics filtering and quality control, 336,853 (67.1%) high-quality sequences belonged to the EcM fungal species, and 165,228 (32.9%) high-quality sequences belonged to the SAP fungal species across all the soil samples. For both guilds, a total of 1457 fungal OTUs were detected in the final rarefied dataset (416 were EcM fungi; 645 were SAP fungi). For ITS gene regions, Good’s coverage was >99% with an average read length of 317 bp. It can be concluded that the sequencing depth was sufficient to assess the diversity and structure of soil fungi across all samples based on the rarefaction curves of the genes.

**Figure 1:**
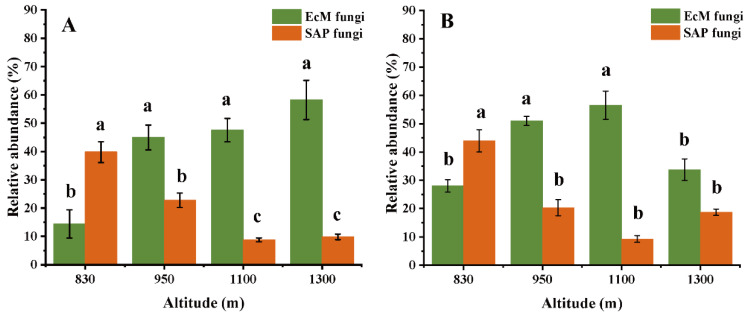
Relative abundances of the EcM and SAP fungal communities at different altitudes in the 0–10 cm (**A**) and 10–20 cm (**B**) soil layers. Different lowercase letters represent significant differences according to Tukey’s HSD test at different altitudes in the same fungal guilds (*p* < 0.05).

In the 0–10 cm soil layer, the relative abundances of EcM and SAP fungi significantly increased and decreased with altitude, respectively. The relative abundance of EcM fungi in the 0–10 cm soil layer at the altitude of 1300 m (58.2%) was significantly higher than that at the altitude of 830 m (14.4%) (*p* < 0.05, Figure 1A). In addition, in the 10–20 cm soil layer, the relative abundance of EcM at 950 m and 1100 m was higher than that at 830 m and 1300 m (*p* < 0.05, Figure 1B). Altitude had a significant effect on the relative abundances of both fungal guilds, but soil depth did not (Figure 1). Additionally, at the OTU level, OTU3356 was the most abundant OTU at both soil depths, with 38.6% (0–10 cm) and 24.4% (10–20 cm) of the total SAP fungal sequences. For EcM fungi, OTU1312 was the dominant OTUs, comprising 13.3% and 14.8% of total EcM reads (Figure S2).

### 3.2. Shared and Unique OTUs of EcM and SAP Fungal among Different Altitude and Soil Depths

The shared and unique OTUs for different altitudinal sites and soil depths were assessed via a Venn diagram, which demonstrated that OTUs differed among samples (Figure S3). The numbers of shared OTUs in the EcM fungal community in the 0–10 cm and 10–20 cm soil layers were 57 and 86, respectively, and those for the SAP fungi were 76 and 97 (Figure S3A–D). The order of the number of unique OTUs for EcM fungi at both soil depths is: 1100 m > 830 m >1300 m > 950 m (Figure S3C,D). In contrast, the unique OTUs in the SAP fungal community exhibited a divergent trend (Figure S3A,B).

### 3.3. EcM and SAP Fungal α and β Diversity along an Altitudinal Gradient in Both Soil Layers

**Figure 2:**
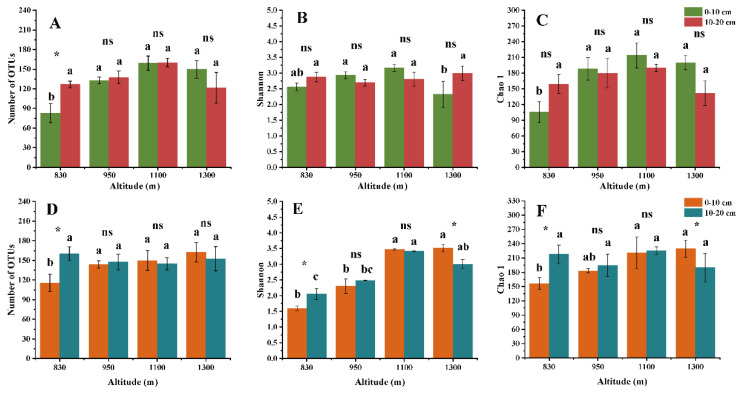
Observed number of OTUs, Shannon and Chao 1 indices of EcM (**A**–**C**) and SAP (**D**–**F**) fungal communities along the altitudinal gradient. (**A**,**D**) Observed number of OTUs; (**B**,**E**) Shannon index; (**C**,**F**) Chao 1 index. Different lowercase letters represent significant differences according to Tukey’s HSD test at different altitudes in the same soil layer (*p* < 0.05). Symbols indicate significant differences according to the Student’s *T* test between different soil layers at the same altitude. ns, not significant; *, *p* < 0.05.

The observed numbers of OTUs and the Chao 1 indices of the EcM and SAP fungi in the 0–10 cm soil layer increased monotonically with increasing altitude (Figure 2). Excluding the altitude of 830 m, no significant difference was observed in EcM fungal alpha diversity between the 0–10 cm and 10–20 cm soil layers (Figure 2A–C). In contrast, the SAP fungal alpha diversity varied significantly at the altitude of 830 m and 1300 m (Figure 2D–F). Notably, the alpha diversity of both assigned fungal guilds (excluding the Shannon index of EcM fungi) exhibited an increasing trend with ascending altitude in the 0–10 cm soil layer (Figure 2).

**Figure 3:**
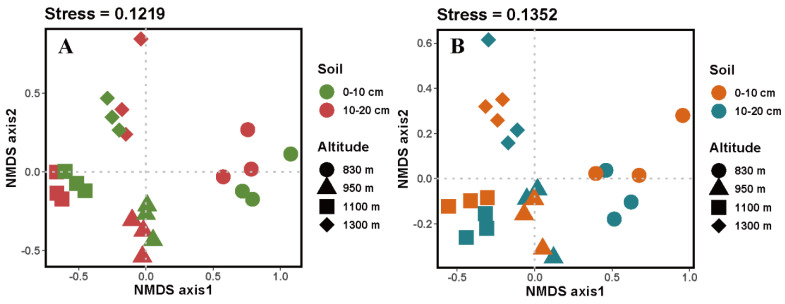
Nonmetric multidimensional scaling ordinations showing EcM (**A**) and SAP (**B**) fungal communities using the Bray–Curtis dissimilarity distance based on OTUs. Numbers after “Altitude” and “Soil depth” are *R*^2^, indicating the variation in EcM and SAP fungal community composition that can be explained by altitude or soil depth, detected using PERMANOVA.

The EcM and SAP fungal beta diversity (community composition) was analysed by Nonmetric multidimensional scaling (NMDS) analysis (Figure 3). The EcM and SAP fungal beta diversities significantly varied among different altitudes. Permutation-based multivariate analysis of variance (PERMANOVA) analyses exhibited significant altitude separation of the EcM and SAP fungal community compositions (EcM fungi, *R*^2^_altitude_ = 0.515; SAP fungi, *R*^2^_altitude_ = 0.620).

### 3.4. Ecological Stochasticity of EcM and SAP Fungal Communities

**Figure 4:**
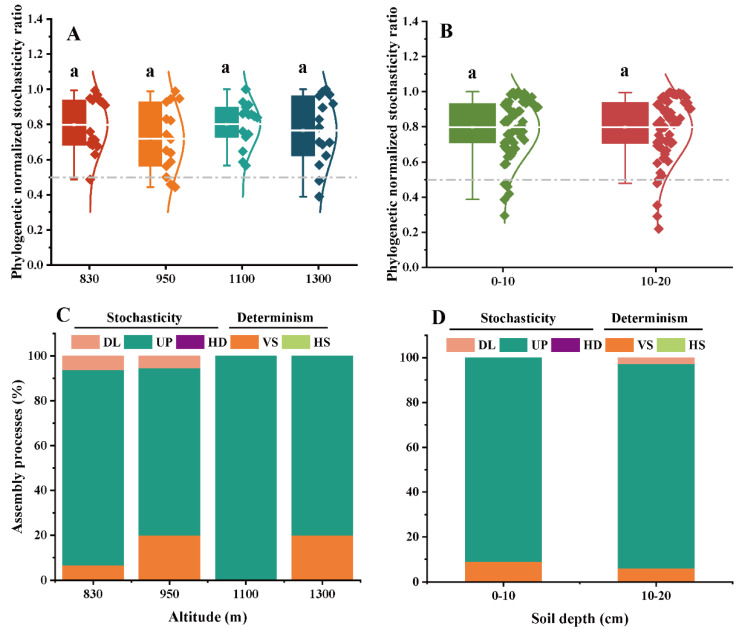
The ecological stochasticity in the potential EcM fungal community assembly is evaluated by the phylogenetic normalized stochasticity ratio (pNST). (**A**) Ecological stochasticity among different altitudes; (**B**) ecological stochasticity between different soil layers. The values < 0.5 and >0.5 represent the deterministic and stochastic assembly, respectively. The relative contributions (%) of the community assembly processes in shaping EcM fungal communities at different altitudes (**C**) and soil layers (**D**). Different lowercase letters denote significant differences at different altitudes (or different soil layers) (*p* < 0.05). Differences between the 0–10 cm and 10–20 cm soil layers were examined using a Student’s *T*-test. Differences among different altitudes were examined using Tukey’s HSD test. HS, homogeneous selection; VS, variable selection; HD, homogenizing dispersal; UP, undominated process; DL, dispersal limitation.

**Figure 5:**
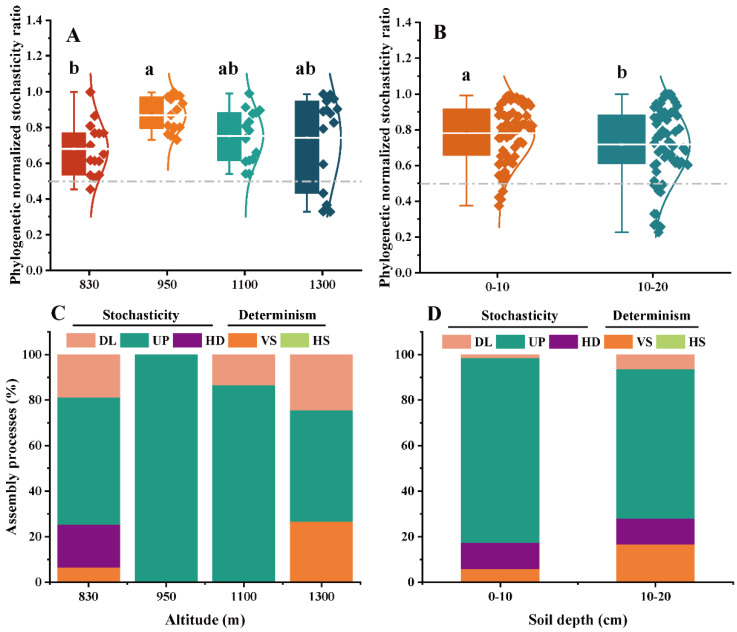
The ecological stochasticity in the potential SAP fungal community assembly is estimated by the phylogenetic normalized stochasticity ratio (pNST). (**A**) Ecological stochasticity among different altitudes; (**B**) ecological stochasticity between different soil layers. The values < 0.5 and >0.5 represent the deterministic and stochastic assembly, respectively. The relative contributions (%) of the community assembly processes in shaping SAP fungal communities at different altitudes (**C**) and soil layers (**D**). Different lowercase letters denote significant differences at different altitudes (or different soil layers) (*p* < 0.05). Differences between 0–10 cm and 10–20 cm soil layers were examined using a Student’s *T*-test. Differences among different altitudes were examined using Tukey’s HSD test. HS, homogeneous selection; VS, variable selection; HD, homogenizing dispersal; UP, undominated process; DL, dispersal limitation.

To further evaluate the ecological processes of the EcM and SAP fungal community assembly, the phylogenetic normalized stochasticity ratio (pNST) based on the taxonomic and phylogenetic metrics was calculated (Figure 4 and Figure 5). The altitude and soil depth significantly affected on the pNST values of the SAP fungal community, not the EcM fungal community (*p* < 0.05, Figure 4A,B; Figure 5A,B; Figure S4). In addition, the stochastic processes dominated the community assemblies of both assigned fungal guilds (Figure 4C,D; Figure 5C,D). The drift process (ranging from 48.9% to 100%) was responsible primarily for the assembly of the EcM and SAP fungal communities (Figure 4C,D; Figure 5C,D; Figure S4). More importantly, a high proportion of variable selection in the SAP fungal community was found at high altitude (1300 m) and in a deep soil layer (10–20 cm) (Figure 5C). Unexpectedly, greater proportions of homogeneous dispersal and dispersal limitation were observed in the community assembly of SAP fungi as compared with EcM fungi (Figure 4 and Figure 5). There was an obvious increase in the dispersal limitation of SAP fungi from the 0–10 cm to the 10–20 cm soil layers (Figure 5D).

### 3.5. Explanatory Factors for Variations in EcM and SAP Fungal Communities

**Figure 6:**
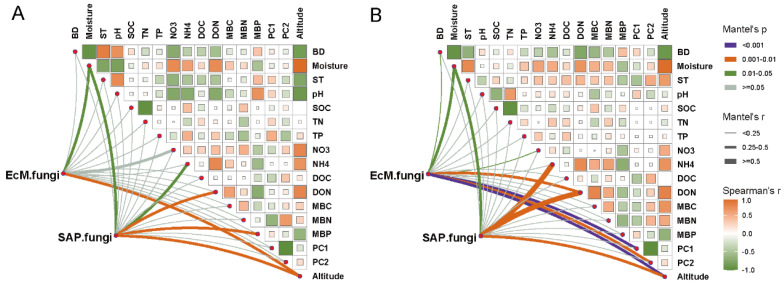
Abiotic and biotic drivers of the EcM and SAP fungal community compositions. (**A**) 0–10 cm soil layer; (**B**) 10–20 cm soil layer. EcM and SAP fungal community compositions (Bray–Curtis distance) were correlated with each environmental or biotic factor by partial Mantel tests. Pairwise comparisons of environmental and biotic factors are shown in the upper-right area, which is represented with a color gradient using Spearman’s correlation coefficients. The edge width represents the partial Mantel’s r statistic for the corresponding correlation, and the edge color indicates that significance is tested following 999 permutations. BD, bulk density; ST, soil temperature; SOC, soil organic carbon; TN, total nitrogen; TP, total phosphorus; NO3, nitrate nitrogen; NH4, ammonium nitrogen; DOC, dissolved organic carbon; DON, dissolved organic nitrogen; MBC, microbial biomass carbon; MBN. microbial biomass nitrogen; MBP, microbial biomass phosphorus; PC1 and PC2, the first and second axis scores of plant community composition.

The differences in soil properties across different altitudes and soil depths have been shown in a previous study (Table S1). Partial Mantel tests were used to correlate the distance-corrected dissimilarities of the EcM and SAP fungal community compositions with environmental variables (Figure 6). The EcM and SAP fungal communities were strongly correlated with the soil moisture and altitude in both soil layers (*p* < 0.05, Figure 6A,B). More interestingly, there was a divergent pattern in the explanatory factors for variations in EcM and SAP fungal communities. The SAP fungal community was predominately affected by NH_4_^+^-N and DON contents, whereas the EcM fungal community was correlated with the plant community composition (*p* < 0.05, Figure 6A,B).

## 4. Discussion

### 4.1. Altitudinal Pattern of Relative Abundance Is Guild-Dependent, but Diversity Is Not

The relative abundances of EcM and SAP fungi exhibited a divergent pattern along the 470 m vertical gradient; that is, higher and lower relative abundances were seen at high altitude sites, respectively (Figure 1). This result is consistent with our first prediction. It indicates an obvious turnover between EcM and SAP fungi along an altitudinal gradient. Phillips et al. [42] revealed that EcM fungi could compete with SAP fungi for nutrient resources and litter decomposition due to more associations with host plants, which suppressed the growth of soil saprotrophs. Moreover, a warming experiment (open top chambers) in the Arctic showed that EcM fungi in low temperature conditions (both air and soil temperatures) had greater richness, whereas SAP fungi tended to adapt more effectively to warm environments [43]. Miyamoto et al. [44] found that in colder locations, EcM fungi showed a narrower temperature range than expected, as well as the highest richness. EcM fungi usually shift their temperature adaptations towards low temperatures, and exhibit ecological tolerance to a low proportion of host plants in cool-temperate forests [13]. In addition, soil fungi generally exhibit a preference for aerobic conditions [45], hence the hypoxia caused by higher soil moisture in high-altitude sites leads to the low relative abundance of SAP fungi.

Despite the mid-domain effect and monotonically decreasing patterns of EcM fungal diversity described by previous studies [12,15], our results showed a consistent altitudinal tendency for EcM and SAP fungal diversity. This finding in contrast contradicts our first hypothesis. Ren et al. [46] found that the EcM and SAP fungal diversities showed similar altitudinal patterns in rhizosphere and bulk soils in Mt. Taibai. In addition, the result of PERMANOVA showed that altitude had a greater effect on both EcM and SAP fungal communities than soil depth. These results further support our first hypothesis. The high-altitude sites have the characteristics of a colder environment and strong physiological stress [47], which may lead to a stronger microbial growth limitation effect than soil depth. Additionally, there were fewer differences in the three alpha diversity indices between the 0–10 cm and 10–20 cm soil layers, which is in line with our previous study regarding soil fungi [35]. Overall, our results suggest that EcM and SAP fungi have a divergent pattern of relative abundance, but a consistent pattern of diversity is detected along an altitudinal gradient in boreal coniferous forests.

### 4.2. Soil Property and Plant Community Were the Driving Factors of SAP and EcM Fungal Communities

Considering the importance of the host and the covariation between the host community and altitude, it is logical to expect that plant community composition has a substantial impact on altitude variations in EcM fungi [14]. In the present study, the results of the partial Mantel tests proved that altitude had a significant effect on both EcM and SAP fungal community compositions, and vegetation community composition strikingly affected EcM fungi in both soil layers, while soil variables (DON and NH_4_^+^-N) were the main driving factors of SAP fungal community compositions (Figure 6). These findings are consistent with our second expectation. Indeed, generally, nitrogen limitation increases with increasing altitude [48], and SAP fungi will be affected by N-related nutrients. In fact, there was considerable *R* value variation in all DON and NH_4_^+^-N contents with altitude. Besides this, the discrepancies observed between EcM and SAP fungal communities could indicate that in cold-temperate locations, EcM fungi may depend more on the host and be less reliant on the edaphic environment than SAP fungi. Shigyo and Hirao [13] demonstrated that EcM fungal richness was primarily affected by host plants, especially regarding the strong impact of the plant traits and EcM fungal community. Thus, more attention should be paid to the coordination between plant economic spectrum and EcM fungi in future research. In addition, besides altitude and plant community composition, soil moisture is a critical factor that influenced both EcM and SAP fungal communities in the present study. Although fewer studies have been undertaken on the relationship between soil moisture and fungal guilds, accumulating evidence demonstrates that soil fungi are highly sensitive to water availability and anoxic conditions [45,49]. Waterlogging and anoxic conditions can be experienced at high elevations due to the occurrence of high rainfall coupled with low evapotranspiration and cooler temperatures [4]. Notably, the soil fungal community composition was mainly affected by the soil moisture, DON and NH_4_^+^-N with varied altitudes, as described by our previous findings [35]. Taken together, the investigation of fungal guilds provides differential and detailed information about the altitudinal patterns of soil fungi.

### 4.3. Stochastic Processes Dominated the Community Assembly of Two Key Fungal Guilds

Prior studies have established that soil microbial community is primarily shaped by niches vs. neutral processes [27,50]. Mounting evidence has proven that biotic and abiotic factors can structure the fungal communities across different ecosystem scales [32,51,52]. Additionally, stochastic processes are considered another primary force governing fungal community assembly in forest ecosystems [33,53]. In the present study, although plant community composition and soil properties significantly affected the EcM and SAP fungal community composition, the community assembly of both fungal guilds along an altitudinal gradient was principally dominated by stochasticity, and especially drift. These results provide some future support for our third hypothesis. Unexpectedly, environmental filtering (host plants or plant community composition) did not dominate the ecological processes of EcM fungal community in our study. A possible explanation for this might be that the geographical distance generates dispersal barriers and reduces the migration rate of fungal propagules [53]. This finding corroborates the ideas of Gong et al. [14], who indicated that stochasticity (e.g., dispersal limitation) may lead to variations in the EcM fungal richness with altitude at Mt. Baima. Wang et al. [54] in a study of pine-dominated forests in cold-temperate regions, found that stochasticity prevailed in the community assembly of EcM fungi. On the other hand, due to the variations in climate, vegetation and soil factors within small-scale habitats, environmental selection may exhibit less importance in determining the local abundance of fungi than dispersal limitation (or drift) [55]. Although fewer investigations have been performed regarding the community assembly of SAP fungi, Schröter et al. [55] revealed that saprotrophic fungi with a low nearest taxon index were significantly controlled by stochastic processes (immigration and loss from the surrounding metacommunity). Soil microbial communities can deviate from neutral behavior as a result of disturbance; deterministic forces will be restored along temporal trajectories, and then the system will regain its original balance [56]. These results therefore need to be interpreted with caution because of the individual sampling time. Further studies are required to explore the season variable as a potential mechanism impacting the community assembly of EcM and SAP fungi. Notwithstanding these limitations, our results suggest a consistent pattern of community assembly of EcM and SAP fungi along an altitudinal gradient, providing a preliminary understanding of the ecological processes of fungal guilds.

## 5. Conclusions

This study provides some novel insights into the altitudinal patterns of diversity and community assembly of EcM and SAP fungi in boreal forests using high-throughput DNA sequencing techniques. Firstly, our results confirm the contrasting tendencies of relative abundance in both fungal guilds. Higher and lower relative abundances of EcM and SAP fungi were observed at high-altitude sites, respectively. In addition, EcM and SAP fungal diversity and community assembly showed consistent patterns along an altitudinal gradient. The community assembly of both fungal guilds was mainly governed by stochastic processes at different altitudes and soil depths. The drift process contributed to a large proportion of ecological stochasticity. However, besides altitude and soil moisture, the plant community composition and soil variables (DON and NH_4_^+^-N) were the most critical factors affecting the fungal community compositions. Together, our findings suggest the altitudinal patterns of EcM and SAP fungal community characteristics in Chinese *Larix gmelinii*-dominated boreal forests. This study strengthens the idea that deciphering the variation in fungal functional taxa along the altitudinal gradient will help expand our knowledge of the connection between microbiota and ecosystem functions. However, it is necessary to conduct further research on the accurate classification of fungal guilds, because the current classification is inadequately understood [5]. Further work is needed to establish a comprehensive understanding of seasonal succession in large-scale studies.

## Data Availability

Not applicable.

## Supplementary Materials

The following supporting information can be downloaded at: https://www.mdpi.com/article/10.3390/microorganisms10101997/s1, Supplementary Table S1. The soil physicochemical properties of surface and subsurface soils in different altitudes. Supplementary Figure S1. Location of the study area (B) and Oakley Mountain (C) in China. Figure A is a schematic view of the studied transects stretching across the *L. gmelinii*-dominated forest tree lines. Supplementary Figure S2. Heat map analyses of EcM (A) and SAP (B) fungal communities across all soil samples. Colors derived from the raw Z-scores were used to identify the relative abundances of the top 50 most abundant classified EcM and SAP fungal OTUs in each sample. Using the average clustering method and Euclidean distances, all samples were grouped in a hierarchical clustering. Supplementary Figure S3. Venn diagram of the number of shared and unique OTUs for EcM (A, B) and SAP fungal community (C, D) under the different altitudes and soil depths. (A, C), 0–10 cm; (B, D), 10–20 cm. OTUs defined at 97% sequence similarity. Supplementary Figure S4. The ecological stochasticity in the potential EcM (A) and SAP (B) fungal communities’ assembly is evaluated by the phylogenetic normalized stochasticity ratio (pNST). The values < 0.5 and >0.5 represent the deterministic and stochastic assembly, respectively. Different lowercase letters indicate significant differences at different altitudes (*p* < 0.05). Differences among different altitudes were examined using Tukey’s HSD test. Differences between the 0–10 cm and 10–20 cm soil layer were examined using a Student’s T-test. *, *p* < 0.05; ns, not significant.
