# Supplementary material for: View from the Top: Insights into the Diversity and Community Assembly of Ectomycorrhizal and Saprotrophic Fungi along an Altitudinal Gradient in Chinese Boreal Larix gmelinii-Dominated Forests
Source: Microorganisms. 2022 Oct 10;10(10):1997. doi: 10.3390/microorganisms10101997 (PMC9607379; doi:10.3390/microorganisms10101997)
Supplement: Supplementary file 1 [file microorganisms-10-01997-s001.zip › microorganisms-1950505-supplementary.pdf]

Supplementary Materials:

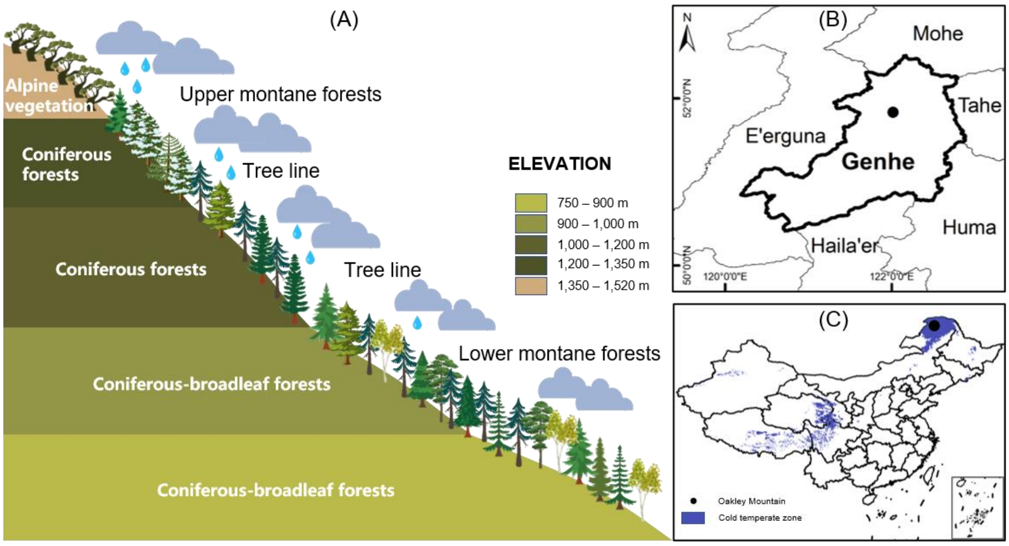

Supplementary Figure S1. Location of the study area (**B**) and Oakley Mountain (**C**) in China. Figure **A** indicates schematic view of the studied transects stretching across the L. gmelinii-dominated forests treelines.

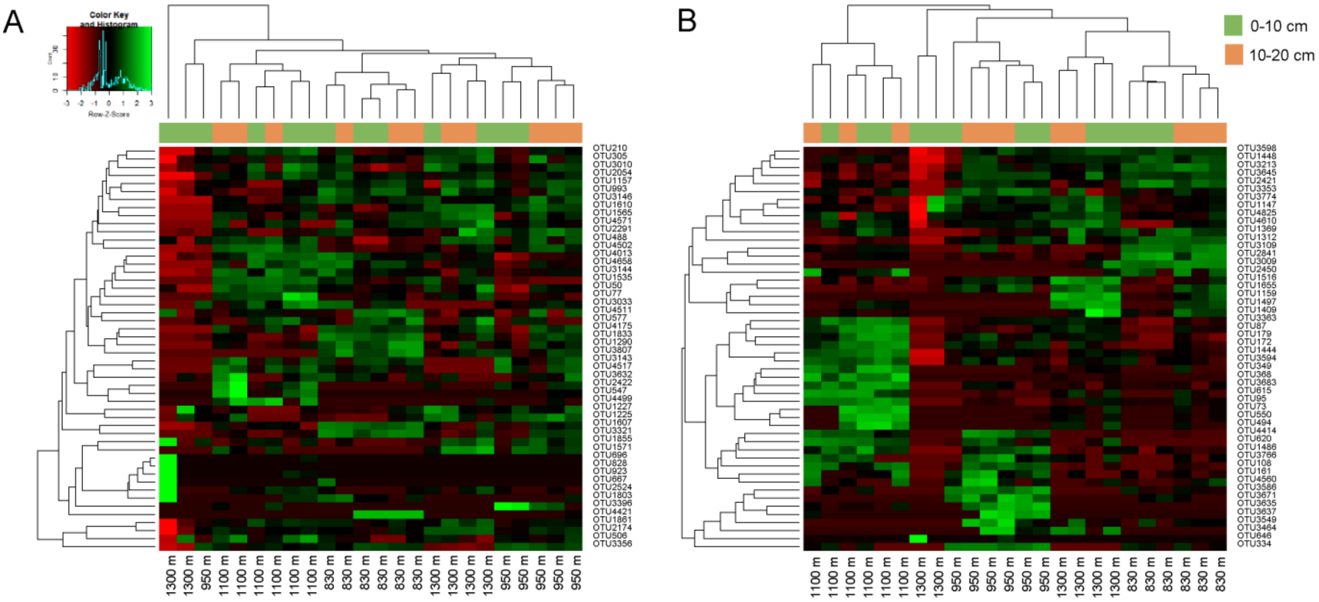

Supplementary Figure S2 Heat map analyses of EcM (**A**) and SAP (**B**) fungal communities across all soil samples. Colors derived from the raw Z-scores were used to identify the relative abundances of the top 50 most abundant classified EcM and SAP fungal OTUs in each sample. Using the average clustering method and Euclidean distances, all samples were grouped in a hierarchical clustering.

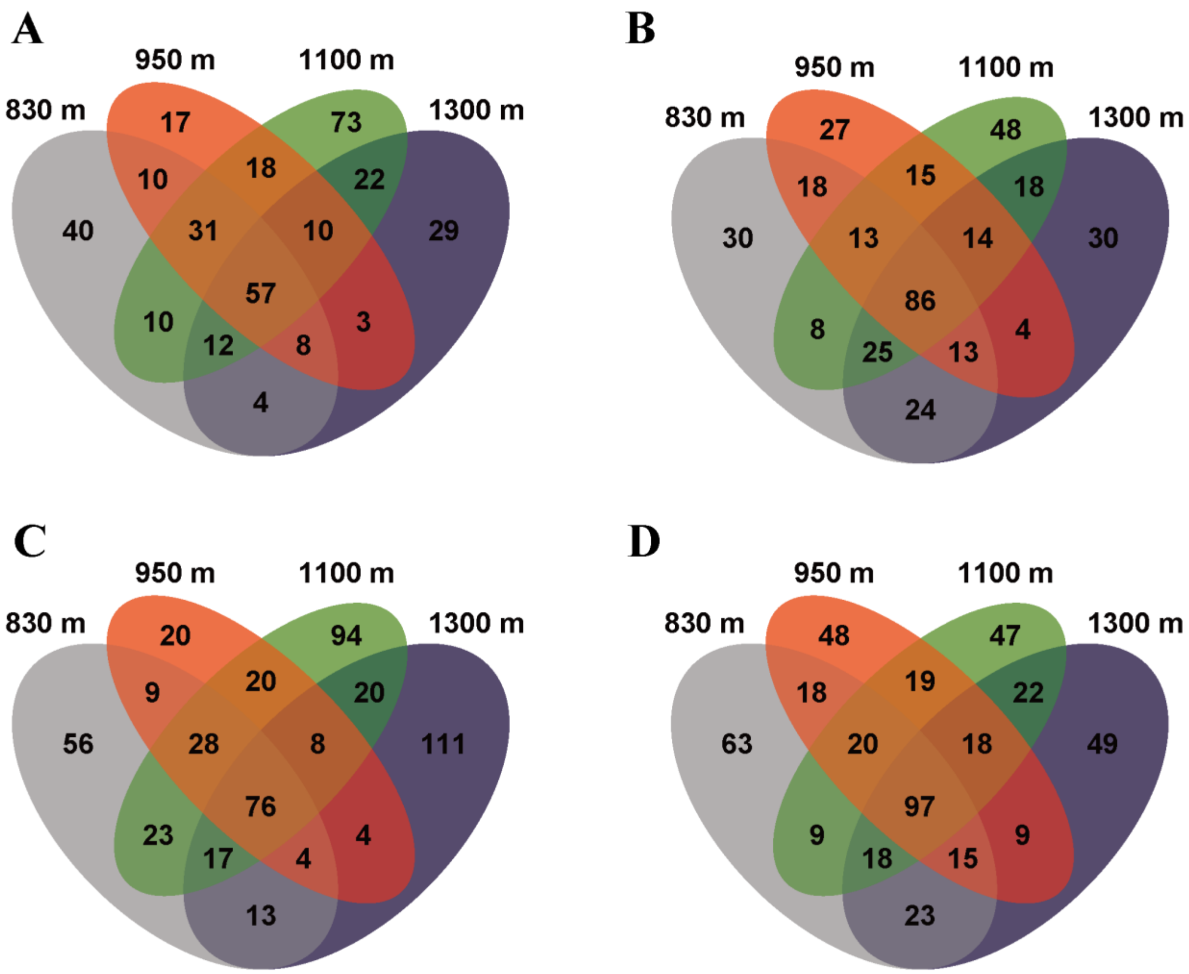

Supplementary Figure S3. Venn diagram of the number of shared and unique OTUs for EcM (**A, B**) and SAP fungal community (**C, D**) under the different altitudes and soil depths. (**A, C**), 0-10 cm; (**B, D**), 10-20 cm. OTUs defined at 97% sequence similarity.

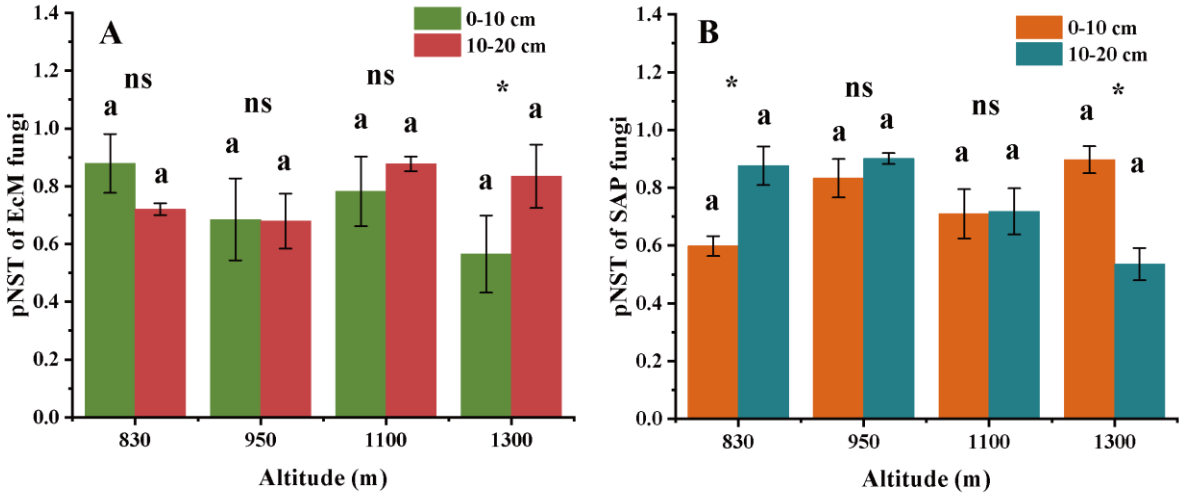

Supplementary Figure S4 The ecological stochasticity in the potential EcM (A) and SAP (B) fungal communities' assembly is estimatedevaluated by the phylogenetic normalized stochasticity ratio (pNST). The value < 0.5 and >0.5 represent the deterministic and stochastic assembly, respectively.

The value of 0.5 is the boundary point between more deterministic (<0.5) and more stochastic (>0.5) assembly. Different lowercase letters indicate significant differences at different altitudes (P<0.05). Differences among different altitudes were examined using Tukey's HSD test. Differences between the 0-10 cm and 10-20 cm soil layer were examined using a Student's T-test. *, P<0.05; ns, not significant.

Supplementary Table S1. The soil physicochemical property for surface and subsurface soils in different altitudes

Soil variables	830 m		950 m		1100 m		1300 m		Two-way ANOVA		
	Surface soil	Subsurface soil	Surface soil	Subsurface soil	Surface soil	Subsurface soil	Surface soil	Subsurface soil	Altitude	Depth	A × D
BD ($g·cm^{-3}$)	0.89±0.13Aa	1.18±0.16Aa	0.63±0.10ABa	0.81±0.08Ba	0.58±0.13ABa	0.64±0.04Ba	0.34±0.02Ba	0.45±0.04Ba	***	ns	ns
Soil moisture (%)	30.75±3.48Ca	20.34±1.31Cb	37.48±1.75BCa	30.7±3.41BCb	47.14±3.85Ba	41.71±5.89Bb	71.01±3.96Aa	58.32±3.55Ab	***	**	ns
ST (℃)	11.12±0.17Aa	7.00±0.58Bb	10.41±0.19ABa	9.02±0.29Ab	10.25±0.35ABa	9.48±0.84Ab	9.01±0.87Ba	9.23±0.28Ab	*	*	ns
SOC ($mg·g^{-1}$)	61.28±2.65Aa	62.08±3.75Aa	65.62±3.26Aa	62.03±0.23Ba	63.14±1.45Aa	61.22±1.91Ba	61.67±2.54Aa	63.83±2.62Aa	ns	ns	ns
TN ($mg·g^{-1}$)	7.02±0.18Aa	6.87±0.32Aa	6.64±0.30Aa	6.97±0.01Aa	6.88±0.15Aa	7.06±0.12Aa	6.97±0.17Aa	6.75±0.20Aa	ns	ns	ns
TP ($mg·g^{-1}$)	0.48±0.05Aa	0.56±0.03Aa	0.19±0.03Ba	0.62±0.04Aa	0.44±0.14Aa	0.34±0.07Aa	0.52±0.17Aa	1.59±0.17Aa	*	ns	ns
pH	4.55±0.02Aa	4.74±0.36Aa	4.31±0.02ABa	4.21±0.18Aa	4.23±0.04ABa	4.28±0.06Aa	4.01±0.45Ba	4.23±0.07Aa	*	ns	ns
NO_3^--N ($mg·kg^{-1}$)	5.39±0.08Ba	5.35±0.25Ba	5.92±0.18Ba	5.62±0.04Ba	5.57±0.09Ba	5.39±0.24Ba	6.97±0.32Aa	6.43±0.31Aa	***	ns	ns
NH_4^+-N ($mg·kg^{-1}$)	63.25±5.23Ba	63.34±5.25Ba	61.27±2.32Ba	58.59±0.53Ba	94.68±4.35Aa	87.72±2.44Aa	88.09±14.84ABa	82.02±3.88Aa	***	ns	ns
DOC ($mg·kg^{-1}$)	163.33±7.96Ba	148.13±4.80Ba	269.60±17.07Aa	295.87±9.12Aa	291.47±7.54Aa	262.80±11.31Aa	213.07±4.92Aa	233.33±12.41ABa	ns	ns	ns
DON ($mg·kg^{-1}$)	15.92±2.31Ba	11.48±1.75Ba	17.57±1.61Ba	15.34±1.22Ba	33.33±2.59Aa	36.57±2.12Aa	32.61±6.74Aa	33.73±1.85Aa	***	ns	ns
MBC ($mg·kg^{-1}$)	456.84±26.86Ba	307.02±11.88Bb	602.81±59.38Ba	538.95±45.01Ba	1565.96±98.72Aa	1515.79±24.21Aa	785.26±54.04Ba	885.26±75.11ABa	*	ns	ns
MBN ($mg·kg^{-1}$)	16.05±1.36Ba	10.11±1.79Ba	60.13±5.40Aa	34.69±1.89Ab	37.78±1.86Ba	36.23±1.15Aa	34.29±1.15Ba	34.59±1.53Aa	*	ns	ns
MBP ($mg·kg^{-1}$)	3.02±0.20Aa	2.82±0.13Aa	2.55±0.21Aa	2.21±0.15ABa	1.31±0.14Ba	1.57±0.18Ba	1.63±0.11Ba	1.41±0.10Ba	*	ns	ns

A: Altitude; D: Soil depth. BD, Bulk density; ST, Soil temperature; SOC, Soil organic carbon; TN, Total nitrogen; NO_3^--N, Nitrate nitrogen; NH_4^+-N, Ammonium nitrogen; DOC, Dissolved organic carbon; DON, Dissolved organic nitrogen; MBC, Microbial

biomass carbon; MBN. Microbial biomass nitrogen. Data with different uppercase letters were significantly difference at 5% level among different altitudes in the same soil layer ($P<0.05$), while different lowercase letters indicate significant differences among different soil layers in the same altitude ($P<0.05$); ns, not significant; *, $P<0.05$; **, $P<0.01$;***<0.001 All data were mean ± standard error (Mean ± SE).
